# Effect of acetylcholinesterase (AChE) point-of-care testing in OP poisoning on knowledge, attitudes and practices of treating physicians in Sri Lanka

**DOI:** 10.1186/1472-6963-14-104

**Published:** 2014-03-04

**Authors:** Bishan N Rajapakse, Teresa Neeman, Nicholas A Buckley

**Affiliations:** 1 College of Medicine, Biology and Environment, Australian National University, Canberra, Australia; 2 South Asian Clinical Toxicology Research Collaboration, University of Peradeniya, Peradeniya, Sri Lanka; 3 Statistical Consulting Unit, Australia National University, Canberra, Australia; 4 Professorial Medical Unit, Prince of Wales Hospital clinical school, Avoca St, Sydney, Australia

**Keywords:** Point-of-care testing, Rural emergency medicine, Resource limited setting, Asia, Laboratory technology, Physician attitudes, Organophosphorus poisoning, Cholinesterase measurement, Oxime therapy

## Abstract

**Background:**

Toxicology and Emergency medicine textbooks recommend measurement of acetylcholinesterase (AChE) in all symptomatic cases of organophosphorus (OP) poisoning but laboratory facilities are limited in rural Asia. The accuracy of point-of-care (POC) acetylcholinesterase testing has been demonstrated but it remains to be shown whether results would be valued by clinicians. This study aims to assess the effect of seeing AChE POC test results on the knowledge, attitudes and practices of doctors who frequently manage OP poisoning.

**Methods:**

We surveyed 23 clinicians, who had different levels of exposure to seeing AChE levels in OP poisoned patients, on a) knowledge of OP poisoning and biomarker interpretation, b) attitudes towards AChE in guiding poison management, oxime therapy and discharge decisions, and c) practices of ordering AChE in poisoning scenarios.

**Results:**

An overall high proportion of doctors valued the test (68-89%). However, we paradoxically found that doctors who were more experienced in seeing AChE results valued the test less. Lower proportions valued the test in guidance of acute poisoning management (50%, *p* = 0.015) and guidance of oxime therapy (25%, *p* = 0.008), and it was apparent it would not generally be used to facilitate early discharge. The highest proportion of respondents valued it on admission (*p* < 0.001). A lack of correlation of test results with the clinical picture, and a perception that the test was a waste of money when compared to clinical observation alone were also comments raised by some of the respondents.

Greater experience with seeing AChE test results was associated with increased knowledge (*p* = 0.034). However, a disproportionate lack of knowledge on interpretation of biomarkers and the pharmacology of oxime therapy (12-50%) was noted, when compared with knowledge on the mechanism of OP poisoning and management (78-90%).

**Conclusions:**

Our findings suggest an AChE POC test may not be valued by rural doctors. The practical use of AChE in OP poisoning management is complex, and a poor understanding of how to interpret test results may have affected its perceived utility. Future research should evaluate the impact of providing both AChE and training in interpretation on clinicians’ attitudes and practice.

## Background

Organophosphorus (OP) insecticide poisoning is responsible for significant mortality and morbidity. The case-fatality of OP self poisoning is high and there are over 200,000 annual deaths worldwide [1].

Toxicology and Emergency medicine textbooks recommend that acetylcholinesterase (AChE) measurement should be performed in all symptomatic cases of OP poisoning where the test is available, as this biomarker may help confirm diagnosis and severity, guide the starting and stopping of oximes by titrating the dose to changing enzyme levels, and may help in guiding patient disposition [2-6]. Some text books recommend checking AChE every 12-24 hours in symptomatic patients [3]. However, accurate laboratory tests require complex collection methods and a lack of availability of reliable point-of-care (POC) laboratory services makes these recommendations difficult to follow [2]. Recent research has validated the use of a POC acetylcholinesterase testing device (Test-mate ChE) for acute OP self-poisoning in rural Sri Lanka, but to date there are no studies to indicate how clinicians would value, and use, such a test should it become available [7]. We were not able to find studies that evaluated the benefit of POC devices in Asian countries, and a deficiency in research surrounding the role of POC testing in a rural hospital setting has also been identified by other researchers [8].

We designed a study, which surveyed the knowledge, attitudes and practices of clinicians in a secondary referral centre who are frequently treating OP poisoning. We looked at what effect exposure to seeing AChE test results had on these parameters. We specifically asked whether clinicians would order such a test if it were available, and how useful it would be in their management of OP poisoning (if at all). Our study intervention was to make AChE results available to clinicians in acute OP poisoning through POC testing, without specific training or education on how to interpret the test results.

Our hypothesis, based upon textbook recommendations, was that AChE tests would be widely ordered and regarded as useful if they were made available. We also hypothesised that doctors with greater experience of the test would report a higher perceived benefit from the test in terms of showing improved knowledge about OP poisoning, guidance of oxime therapy, and the facilitation of early discharge of patients with mild symptoms.

This study aimed to assess the knowledge, attitudes, and practices amongst a range of treating clinicians who manage high volumes of OP poisoned patient, and who did not generally have access to AChE results. We also aimed to report changes that occurred in relation to the introduction of a point-of-care AChE device [7].

## Methods

The University of Peradeniya Ethical Review committee approved the study, and consent was implied by participation in a paper survey.

### Selection of doctors

The study targeted the practicing doctors who worked in the General Medical ward and intensive care ward of a secondary referral centre (with approximately 800 hospital beds) in a rural Sri Lankan setting. All levels of the medical hierarchy including Consultants, Senior House officers (SHO’s), Medical officers (MO’s), and house officers (HO’s) were included in the study.

### Intervention

The intervention consisted of the provision of AChE results from OP poisoned patients, over a 13 month period, to treating clinicians. Clinicians were surveyed at beginning and end in order to capture respondents with a range of experience of the seeing AChE test results (see Additional file 1: Figure S1).

#### Measurement of AChE results

Blood samples were taken from all consenting OP poisoned patients admitted to the medical wards and intensive care unit by trained research assistants according to a study protocol and the results were added to the patient record so that clinicians could use this information if they desired.

**Figure 1:**
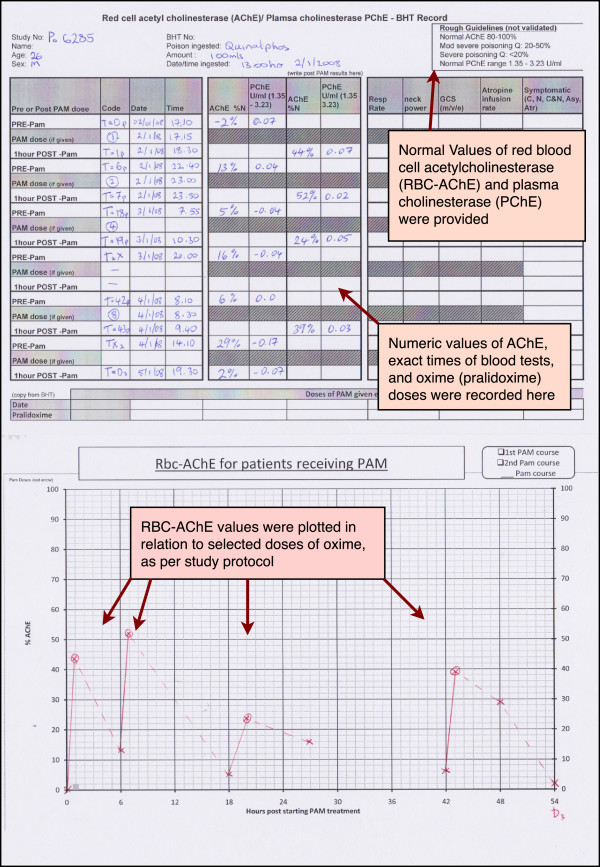
**Shows the method of making AChE levels from OP poisoned patients available to treating clinicians (study intervention).** Red blood cell acetylcholinesterase (AChE) and plasma cholinesterase (PChE) results were presented to clinicians via results sheet consisting of a table and graph which was added to patient record.

The RBC-AChE and PChE levels were measured, using the Test-mate ChE point-of-care device, before and after doses of oxime in patients who were on this therapy. These results were graphed to highlight any relationship to doses given (see Figure 1), in a similar fashion to what has been recommended in the literature [5].

#### AChE testing protocol

The test was made available in the hospital for 13 months during the time of the survey. A specific protocol guided the frequency and timing of blood tests in relation to oxime doses (see Additional file 2). Symptomatic patients who were treated with oximes had blood tests taken pre and post doses of oxime during the first 48 hours, and a pre and post dose test was taken on a daily basis thereafter. Symptomatic OP poisoned patients who weren’t treated with oximes were tested at the same time points, omitting the post oxime measurement.

Asymptomatic patients had an initial blood test followed by a daily blood test thereafter. Patients with unknown poisoning were treated as described depending on whether they were symptomatic or not, and whether they were being treated with oximes or not. After 5 days of admission the frequency of blood testing was reduced to alternate day testing.

#### Management of OP poisoned patients on whom tests were performed

Internal medicine physicians were responsible for the management of all pesticide poisoned patients who were treated according to local practice. Symptomatic OP poisoned patients were generally resuscitated with atropine via an intravenous bolus followed by infusion, with the dose titrated to clinical response. Oximes were sometimes prescribed depending on the preference of the treating physician.

### Study endpoints

#### Survey of treating doctors

A self reported survey (Additional file 3) was distributed to all the doctors including an information sheet explaining the confidentiality of the data that would be obtained.

The survey had 3 components:-

1) 25 True/false statements (worth 1 mark each) organized into 5 questions on knowledge of OP poisoning, use of oximes and AChE testing

2) Short answer questions assessing attitudes towards AChE testing and choice of oxime therapy

3) Scenario based questions assessing clinical practice regarding ordering AChE tests, oxime therapy, and patient discharge.

The principal investigator delivered the questionnaire personally to each doctor in the sampling frame of 40 doctors responsible for treating poisoned patients. They were instructed to complete and return the questionnaire as soon as possible.

#### True/false knowledge questions

The knowledge component comprised 5 areas each covered with 5 true/false statements. Each correct answer was given one mark and each incorrect answer a negative mark resulting in a maximum score of 5 and a minimum score of -5 for each of the 5 areas, which was then converted into a percentage. The questions tested knowledge on the mechanism of OP pesticides toxicity, the inhibition of AChE, types of biomarker in OP poisoning, the clinical correlation of biomarker levels in poisoned patients and the response of AChE to oximes (Additional file 3 2.1). The level of difficulty was aimed at ‘expert’ level (ie. a level that a toxicologist could be expected to know).

#### Short answer questions on experience and attitudes

A short answer question format was used to assess the doctors’ prior experience with treating OP poisoned patients, number of AChE test results seen, and perceived usefulness of the AChE test (Additional file 3 2.2). The doctors usual practice regarding the dose and duration of oximes therapy in mild, moderate and severe OP poisoning was recorded in this section.

#### Scenario evaluation of attitudes and practice in cases of OP poisoning

The last part of the survey consisted of four commonly encountered patient scenarios: two scenarios with severe poisoning, and florid cholinergic signs, and two scenarios with mild poisoning, either receiving or not receiving oximes (Additional file 3 2.3). These scenarios were based upon commonly encountered patients. The scenarios assessed whether oximes would be prescribed, and whether an AChE test would be ordered (and thus considered beneficial OP poisoning management) at different time points in course of the patient admission. Survey questions also explored the willingness to discharge a mildly poisoned patient earlier than the standard 4 days of inpatient observation.

#### Clarifying statements as an adjunct to survey data

We provided respondents with the opportunity to clarify their choices with free text. Headings such as “Comments…”, or prompts like “Why/Why not?” would follow questions that required either a binary or categorical answer such as “Do you think an acetylcholinesterase level (AChE) will be useful in helping guide treatment with oximes?” (see Additional file 3 2.2 & 2.3 for examples of survey).

### Survey analysis

The study was initially designed to compare survey responses before and after exposure to the intervention (which was participation in an observational study providing doctors with bedside AChE results). Because some doctors reported prior experience with AChE tests, and some respondents reported having no experience of seeing AChE levels after the intervention we decided to analyse the survey respondents as a cross sectional sample categorizing respondents according to level of experience with seeing AChE test results, and comparing the groups. Respondents were divided into three groups (no tests, 1-5 tests, or 5-20 tests) based on the number of AChE test they reported they had seen, which was asked in the first section of the survey. Three doctors completed the survey twice during the study period and we only used the first survey response in this group so that the data we analysed was uniform with regards to not having previously completed a survey (see Additional file 1: Figure S1).

### Statistical tests

The mean scores from the knowledge questions were compared using the oneway ANOVA test. The categorical answers to the survey questions were analysed using the Kruskal Wallis test where the data was ordinal, and the Fisher’s exact test where the data was nominal. The answers for some questions were coded as ordinal data where some authors could have viewed it as nominal data. For example, when respondents were asked if AChE could guide oxime therapy and the possible answers were “no”, “not sure” or “yes”, because in this context “not sure” represented a point mid way between “yes” and “no”. Also in the answers where respondents would select the dose of oxime that would be prescribed, the categorical choices were an increasing dose so we coded their responses as an ordinal variable. The scenario data which comprised multiple responses from doctors were analysed using generalized estimating equations (GEE). Associations between patient risk factors and the decision to order AChE test were expressed using odds ratios. All statistical calculations were performed on STATA version 12, and graphs drawn on Prism version 6.

## Results

The 22 doctors who were included in the analysis consisted of both senior and junior medical staff from departments of medicine (68%) and intensive care (32%), and they reported treating on average 51-100 cases of OP poisoning per year (see Table 1). Their experience of seeing the point-of-care AChE test results ranged from never having seen a test previously (ie ‘0 tests’) in 11 participants, to having seen ‘1-5 tests’ in 7 participants, and ‘5-20 tests’ in 4 participants.

**Table 1:** Survey respondent characteristics

	***Number of respondents (%)***
***Seniority***	
Consultant	3 (14%)
House officer	11 (50%)
Medical officer	5 (23%)
Senior House officer	3 (14%)
***Specialty***	
Medicine	16 (72%)
Intensive Care	6 (27%)
***No of OP poisoned patients seen***	
Less than 5	2 (9%)
5 to 20	5 (23%)
21 to 50	5 (23%)
51 to 100	5 (23%)
greater than 100	5 (23%)
***Exposure to AChE tests***	
Zero	11 (50%)
1 to 5	7 (32%)
5 to 20	4 (18%)
21 to 50	0 (0%)
51 to 100	0 (0%)

### Knowledge

**Figure 2:**
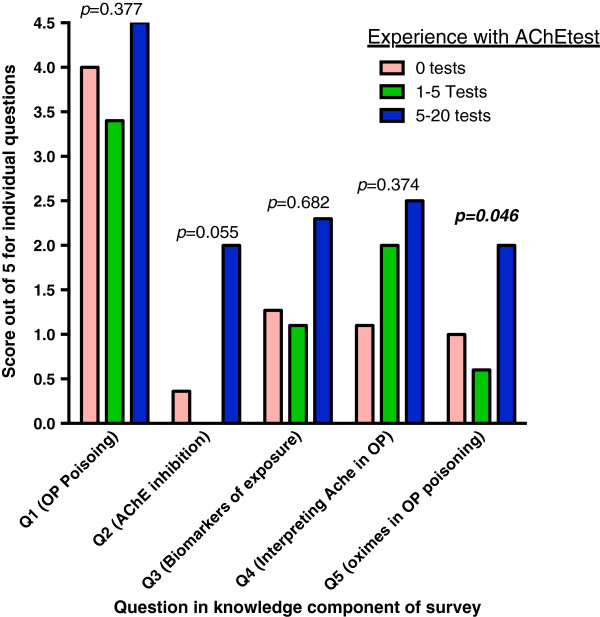
**Knowledge scores for questions (based on answers to true/false statements).** This column graph shows the differences in scores by level of experience with the AChE test.

Those with most experience of AChE test results (eg. 5-20 tests) had the highest knowledge with a significant increase noted in the total score (*p* = 0.034, see Figure 2). This was most marked in the questions to do with “biomarkers of exposure”, “interpreting AChE in OP”, and “oximes in OP poisoning”, but the increase only reached statistical significance for question 5 on “oximes in OP poisoning” (*p* = 0.046).

### Attitudes: AChE in OP poisoning management

**Figure 3:**
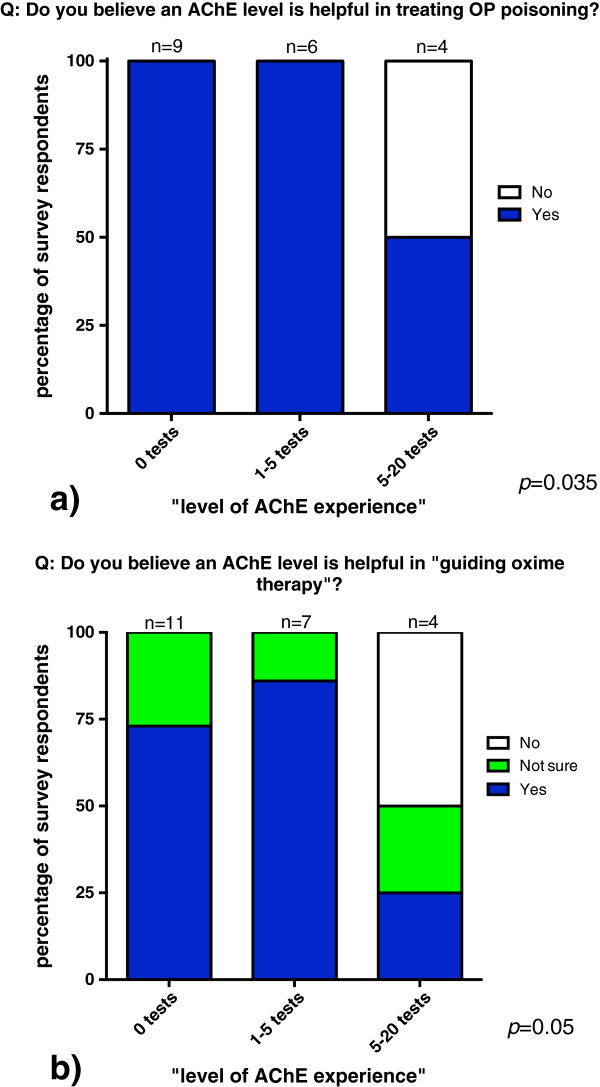
**Attitudes towards AChE testing in organophosphorus management.** The perceived value of an AChE level **(a)** in the treatment of organophosphorus poisoning, and **(b)** in the guidance of oxime therapy is shown according to level of experience with the test.

The perception that AChE test was useful in managing OP poisoning was 100% amongst respondents with no AChE test experience (0 tests) and minimal experience (1-5 tests), but was significantly less (50%) in respondents that had the most experience of seeing the most test results (5-20 tests) (*p* = 0.035, Figure 3a, Table 2).

**Table 2:** Attitudes towards oxime therapy and AChE testing †

**Question**	**Response**	**Level of AChE experience**			***p *** **value**
		**"0 tests" n (%)**	**"1-5 tests" (%)**	**"5-20 tests" n (%)**	
What dose of intravenous pralidoxime would you prescribe to a **severely symptomatic** patient?	None	0 (0%)	1 (14%)	0 (0%)	0.008
	1 g 6 hourly	10 (91%)	6 (86%)	1 (25%)	
	2 g bolus + 500 mg continuous IV infusion	1 (9%)	0 (0%)	3 (75%)	
For what duration would you give the above dose?	None	0 (0%)	1 (14%)	0 (0%)	0.089
	24 hours	1 (11%)	0 (0%)	0 (0%)	
	48 hours	6 (67%)	5 (71%)	0 (0%)	
	Other time period	2 (22%)	1 (14%)	3 (100%)	
What dose of intravenous pralidoxime would you prescribe to a **mildly symptomatic** patient?	None	1 (9%)	4 (57)	1 (25%)	0.105
	1 g 6 hourly	10 (91%)	3 (43)	2 (50%)	
	2 g bolus + 500 mg continuous IV infusion	0 (0%)	0 (0%)	1 (25%)	
For what duration would you give the above dose?	None	1 (11%)	4 (57%)	1 (33%)	0.116
	24 hours	0 (0%)	0 (0%)	1 (33%)	
	48 hours	7 (78%)	3 (43%)	1 (33%)	
	Other time period	1 (1%)	0 (0%)	0 (0%)	
What dose of intravenous pralidoxime would you prescribe to an **asymptomatic** patient who is not getting atropine?	None	6 (64%)	7 (100%)	4 (100%)	0.097
	1 g 6 hourly	4 (36%)	0 (0%)	0 (0%)	
	2 g bolus + 500 mg continuous infusion	0 (0%)	0 (0%)	0 (0%)	
For what duration would you give the above dose?	None	5 (56%)	4 (100%)	4 (100%)	0.435
	24 hours	2 (22%)	0 (0%)	0 (0%)	
	48 hours	2 (22%)	0 (0%)	0 (0%)	
	Other time period	0 (0%)	0 (0%)	0 (0%)	
Do you think an acetylcholinesterase level (AChE) will be helpful in guiding treatment with oximes?	Yes	8 (73%)	6 (86%)	1 (25%)	0.051
	Not sure	3 (27%)	1 (14%)	1 (25%)	
	No	0 (0%)	0 (0%)	2 (50%)	
Is this test was available and affordable. Would it be useful in treating OP poisoning?	Yes	9 (100%)	6 (100%)	2 (50%)	0.035
	No	0 (0%)	0 (0%)	2 (50%)	

† The total number of answers for each question do not always add up to 22 as some respondents omitted answering questions.

However, the two respondents who stated the test was “not useful” qualified their answers with the following statements suggesting a mixed impression about the utility of the test;-

“Not necessary to manage OP poisoning but useful to identify (diagnosis)”

“AChE level is not related to the amount of poison or the clinical symptoms but is useful in unknown poison management”

The AChE test was noted to be helpful in guiding oxime therapy in 73% and 86% of respondents with no experience, or minimal experience, but this proportion was significantly less (25%) amongst respondents with the most experience of seeing AChE test results (*p* = 0.05, see Figure 3b, Table 2).

Respondents who reported that the test was helpful in guiding oximes clarified their choice with comments that valued the role of AChE in assessing severity of poisoning, and the titration of AChE levels with oxime administration;-

“I think acetylcholinesterase is a fairly reliable method of assessing the level of poisoning”

“need to check whether the AChE level is up or down with oxime”

Others raised concerns regarding the interpretation of test result despite stating that they thought it was helpful;-

“Yes. But I am not sure of a cut off point to decide on giving oxime - Evidence is needed on this”

“I think it is useful in guiding the effectiveness of oximes. I don’t think there is a symptomatic correlation”

Conversely, respondents who did not believe that the test was helpful in guiding oximes, expressed concern about the clinical correlation of AChE in OP poisoning;

“(AChE) level does not correlate with clinical symptoms of the patient”

as well as valuing clinical assessment over biomarker evaluation;

“the most important thing is whether the patient is symptomatic or not”

The oxime dose and duration in relation to severity of poisoning, and the range of oxime prescription patterns amongst survey respondents is shown in Table 2.

**Figure 4:**
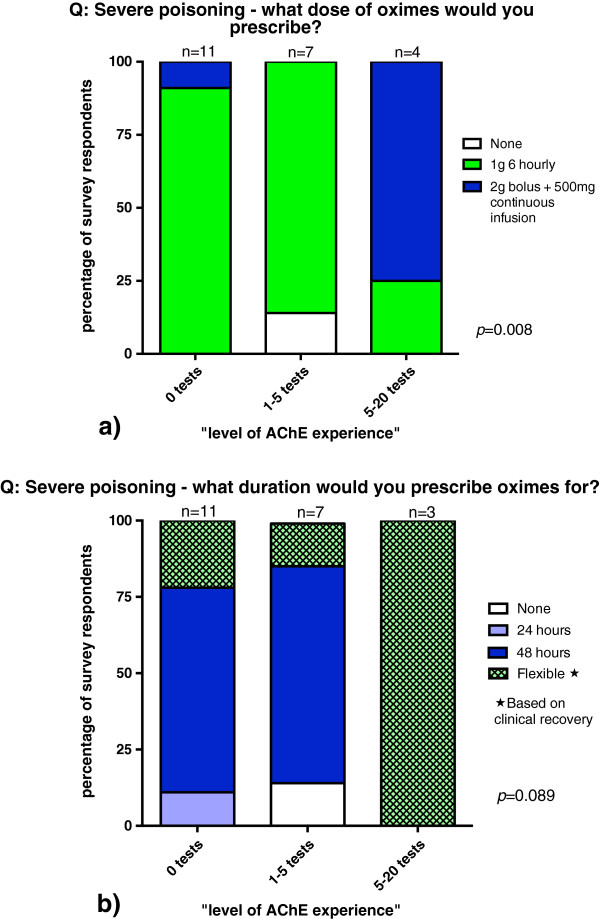
Attitudes towards oxime dose (a) and duration (b) in a case of severe OP poisoning, show by level of AChE test experience.

The majority (75%) of the group with most experience chose a dosing regime of “2 g intravenous bolus followed by 500 mg/hour intravenous infusion”, compared with 9% and 0% of respondents from the subgroups with no experience and minimal experience (*p* = 0.008, see Figure 4a). These latter two subgroups chose to use a 1 g boluses every 6 hours, 91% and 86% of the time, instead.

The subgroup with most experience also chose a “flexible” duration of therapy contrasting those with no experience or minimal experience, who chose 48 hours of therapy in 67% and 71% of responses respectively (*p* = 0.089, see Figure 4b).

The comments for this section clarified that the popular “flexible” duration of therapy in the group of respondents with most experience was related to clinical recovery, (see examples below);-

“*Until the patient is asymptomatic or off atropine*”

“Until the patient gets rid of the OP effects clinically”

No respondents made specific reference to the concept of an AChE level guiding either the “dose” or “duration” of oxime therapy in the clarifying comments for this section.

### Scenario analysis: ordering AChE at different time points in admission

**Figure 5:**
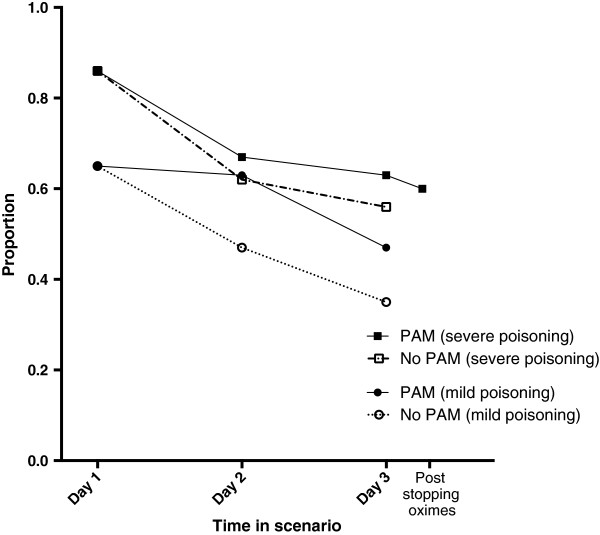
**Practice; showing proportions of respondents who would order an AChE test in different clinical scenarios.** The effect of time, clinical severity, and concurrent oxime therapy on willingness to order an AChE test is demonstrated.

“Time” (since admission) was a factor affecting whether respondents would order an AChE test, with a lower propensity for ordering an AChE test each subsequent day in the hospital admission (OR 0.85 [0.79-0.91], *p* < 0.001, see Figure 5). AChE was ordered most frequently on admission (ranging from 86% to 65%, depending on severity and concurrent oxime therapy), and a progressive decline was noted during the following 3 days of hospital admission. Conversely there was an increased propensity for ordering an AChE test with greater severity of poisoning (OR 1.22 [1.10- 1.37], *p* < 0.001). There was a trend for increased propensity of ordering an AChE test in scenarios where oxime therapy was concurrent, however the influence of “oxime therapy” was not statistically significant (OR 1.09 [0.96-1.25], *p* = 0.182). We noted a lower propensity for ordering AChE in those with the most experience compared those with no test experience, a difference which approached statistical significance (OR 0.78 [0.61-1.00], *p* = 0.052), when level of experience with seeing AChE test was considered.

### Scenario analysis: ordering AChE and guidance of oxime therapy

A minority of clarifying comments in this section of the survey stated that ordering of AChE would help with making a decision about oxime therapy;-however, such comments were not offered by the majority or respondents, nor were they offered over the range of time points in the scenario.

“to decide whether to prescribe pralidoxime or not” [Severe poisoning, oximes, Day 2]

“can obtain a relative idea of the proportion of aging” (no specific reference to oximes; but reference to ‘aging’ may be implied to relate to the effectiveness of oxime therapy) [Severe poisoning, no oximes, Day 3]

We noted a trend for higher proportions ordering an AChE test after oximes were stopped, in the scenario of severe poisoning, when comparing respondents with minimal and most experience (100% and 75% respectively), with those with no experience (37%, see Additional file 4: Figure S2). This observation suggests the use of AChE in guiding a decision to re-starting oximes, however, the difference in proportions was non significant (*p* = 0.450). Furthermore, the clarifying comments did not clearly identify this as a reason for AChE being ordered at this time point in any of the respondents, regardless of their level of experience.

One comment indicated that AChE may be used in this way by saying the reason for ordering the test post stopping oximes was;but there was a lack of detail about what action would be taken if the AChE enzyme levels had decreased, and it is uncertain whether the respondent would use the comparison to restart oxime therapy.

“to compare with initial enzymes”

### Scenario analysis: early discharge and ordering AChE

If AChE was perceived as helpful in facilitating early discharge one would expect a high proportion of respondents ordering an AChE test on day 2 or day 3 in the scenarios of a mildly poisoned OP patient, and comments that showed a link between the use of an AChE level to aid the decision to discharge a patient.

**Figure 6:**
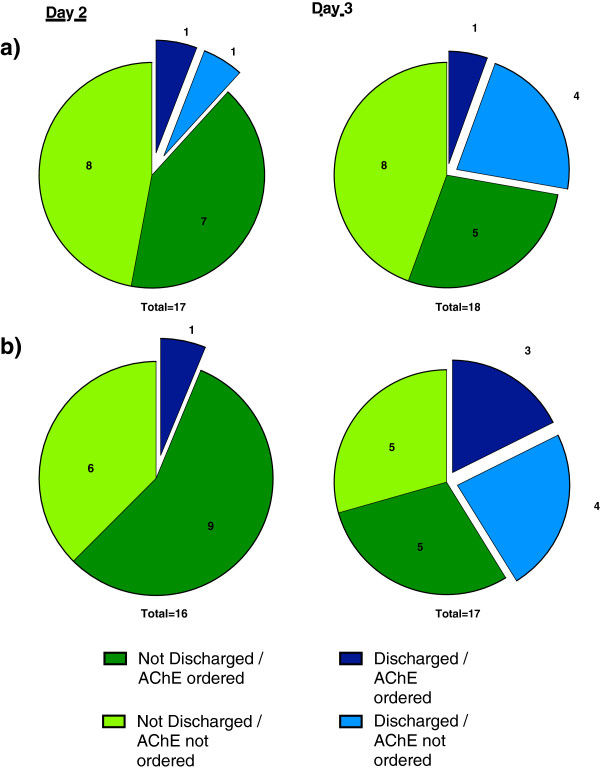
Association of ordering an AChE test with the early discharge of a mildly poisoned patient who has a) initially received oximes, or b) initially not received oximes.

This was not the case as respondents suggested they would order an AChE test on only 38% of occasions (6/16) where they said they would discharge a patient home, and there was no association between the decision to order an AChE test and the decision to discharge (see Figure 6). We also noted that in general a low proportion (50%) of respondents would opt to discharge a mildly symptomatic patient home on either day 2 or day 3 post admission.

However, two respondents made the following clarifying comments as reasons for ordering an AChE test;which suggests that the role of AChE levels in guiding disposition decisions was considered by at least a minority of respondents.

“can discharge the patient early”

“it would decide in keeping or discharging the patient”

### Other reasons for ordering AChE based on comments

Respondents qualified their answers with clarifying comments for just under half (48%) of the scenario questions in favor of the decision to order an AChE test, with 48% (see Additional file 5: Figure S3), providing some insight into the thinking behind ordering and not ordering an AChE test.

Many answers supported perceived value of AChE testing having a role in diagnosis of OP poisoning, and gauging severity;and in guiding oxime therapy;

“(useful) in cases of unknown pesticide poisoning for guiding treatment on certain occasions”

“I think acetylcholinesterase is a reliable method of assessing level of poisoning”

“it will be an indicator showing the degree of poisoning”

“Titrate the pralidoxime dose frequency with the enzyme level in the plasma”

“when to start oximes, When to stop oximes”

“(with measurement of AChE) unnecessary treatment can be avoided”

However, the details of specifically how oxime doses would be titrated were absent as highlighted in the previous section.

Many statements that were against the use of AChE in management of OP poisoning highlighted the sentiment that “clinical assessment” should take precedence over biochemical guidance;and other comments raised concerns about the potential wastefulness of such an investigation;

“Level does not correlate with clinical symptoms of the patient”

“Most important thing is if the patient is symptomatic or not”

“this will not change patient management, it will waste money”

## Discussion

The literature states that the AChE levels can help guide OP poisoning management, oxime therapy and disposition decisions, and our study found that “overall” a high proportion of surveyed doctors valued ordering AChE test in accordance with these recommendations. However, paradoxically we noted that doctors with more experience of seeing test results through the study intervention were less likely to value the AChE test in guiding OP poisoning management and oxime therapy, and there was no suggestion it would be used to facilitate early discharge. Respondents raised a number of general concerns about a lack of correlation between test results and the clinical picture, and a perception that ordering a test would be a waste of money when compared to the standard practice of clinical observation.

The experience of seeing test results was associated with improved knowledge scores across all domains. These scores were highest for knowledge in clinical management of OP poisoning (78% improving to 90% post intervention), contrasting the lower scores for questions related to biomarkers and the use of oximes (12-20% improving to 40-50% post intervention). A relative lack of knowledge about the interpretation of biomarkers in guiding general management and oxime therapy may have been one factor explaining our unexpected findings.

### Difficulty in interpretation of results: Pitfalls in AChE monitoring

AChE is being increasingly recognized as a complex test with regard to the interpretation of levels in the context of acute OP poisoning. Difficulty with interpretation of AChE levels arises for several reasons including a variation in cholinesterase inhibition from different types of OP agents [9], the wide normal range for both kinds of AChE (plasma cholinesterase and red cell cholinesterase), irreversible inhibition (“ageing”) by a proportion of the enzyme, and the fact that inhibition can be non specific due to other factors that can reduce AChE levels such as concurrent chloroquine therapy, or conditions like pernicious anaemia [1,3]. Re-inhibition of AChE may occur when an oxime is discontinued and there is a residual poison load. Thus, in such situations it may be dangerous to discharge based on early recovery of AChE. Our study found that doctors generally would not use AChE to support discharge decisions, however, the explanations provided suggested that such nuanced considerations were not relevant. Further, comments suggested clinicians found it challenging to negotiate the pitfalls in AChE measurement highlighted in textbooks.

### Lack of specific decision rules for AChE guidance

The lack of knowledge regarding AChE interpretation may in part be explained by a lack of precise values of AChE informing decisions rules in OP poisoning management [2-6]. This gap in the literature was apparent on further examination of recommendations regarding the use of AChE in guiding oxime therapy and disposition decisions in particular. In relation to re-starting oximes, for example, one text quoted “further deterioration of cholinesterase activity should be treated by reinstituting a pralidoxime infusion, even though the patient may still be asymptomatic” [3], without providing numeric qualification on the ‘degree of inhibition’ that should lead to action. The challenge of interpretation of such advice, and a similar style of advice in other textbooks was well described in the comments of one respondent in particular from the scenario section of the survey who wrote;

“*I am not sure of a cut off point to decide on giving oxime - Evidence is needed on this”*.

There is a complete lack of evidence regarding the practical use of AChE in guiding oxime therapy. While some texts have suggested graphical representations of the titration of oximes with AChE levels [7], these only provide guidance on principles of antidote titration rather than specific levels that can be referenced in an algorithmic fashion. More sophisticated laboratory test methods exist to support decision making and oxime therapy; for example the in vitro measurement of response to oximes and estimation of the reactivatable RBC-AChE enzyme [10,11]. However, the practical employment of these methods in POC tests has not yet been described or validated.

There is also little data to guide interpretation of AChE for it’s role in of supporting disposition decisions. Here many texts recommend discharge in association with other features like cessation of a need for further antidote therapy, clinical improvement and “stable” or “minimally depressed” cholinesterase activity [2,3].

### Clinical correlation of AChE, and outcome

On the other hand, the evidence on how RBC-AChE can provide guidance about diagnosis and severity of poisoning has been more clearly described, with extent of inhibition correlated with clinical findings [7,10,12-16]. However, even the when specific ranges of AChE are known, the application of this information in a clinical setting may be complex, as it requires clinicians to incorporate an understanding of the potential pitfalls that can be encountered. Variations in AChE inhibition from different agents may lead to “some patients presenting highly symptomatic after minimal reduction in cholinesterase, whilst others can be asymptomatic after losing 50% of activity” [3].

Thus AChE appears to be a test where background factors, such as understanding it’s role in pathophysiology of poisoning, are important in making decisions about treatment, but there is no specific guidance available for doctors.

This point can be illustrated by contrasting the use of AChE in OP poisoning management with the use of peak expiratory flow rate (PEFR) in management of acute asthma. PEFR can also be used for guiding diagnosis, severity and disposition decisions in emergency departments. The difference is that published decision rules for the use of PEFR in asthma management exists [17,18], whilst similar decision rule research and evidence based guidelines for the use of AChE in OP poisoning management is lacking.

### Study intervention provided without education or training

No attempt was made to train doctors on how to use the test, the results were presented in a format that would allow the AChE test to show benefit, such as by graphing pre and post oxime doses (see Figure 1) to facilitate guidance of oxime therapy. Given national recommendations [4] were available for AChE interpretation we expected the test to be used widely in clinical treatment. Our results highlight the effects of introducing a complex test without specific training in how to interpret test results.

It is interesting that knowledge increased without didactic teaching. One possible explanation is that by seeing the tests results doctors had an increased awareness about the mechanisms of OP poisoning (inhibition of the AChE enzyme), and the use of oximes to regenerate inhibited AChE.

A qualitative study assessing the introduction of a different POC test reported that some clinicians were more likely to use certain tests if they had recent formal education in the domains surrounding the test [8]. The difficulties surrounding the interpretation of AChE in the context of OP poisoning, that have been observed in the current study, emphasise the importance of training doctors who may use the test on the assay’s capabilities and limitations. Such training should include appreciation of the pitfalls of collection, measurement, interpretation of AChE results in the context of guiding oxime therapy and facilitating discharge.

Educational interventions are likely to affect attitudes and practices, and future studies should therefore incorporate concurrent education into the assessment of new POC tests.

### Limitations

Our initial planned design was pre-post analysis but because of the high turnover of doctors in the study intervention wards, we ended up with data suited to a cross sectional survey, comparing subgroups by “level of experience with seeing AChE test results”. Small numbers in these sub groups, despite a fairly good response (26 out of 40) was a limitation. However, the effect of the study intervention was large enough to result in significant differences between subgroups in some domains.

### Less “AChE test experience” than expected

Whilst we recorded a range of AChE experience amongst survey respondents we expected the range to be broader, with some doctors potentially seeing up to 100 test results (20 tests was the highest reported experience in our study). A total of 81 patients had AChE levels measured during the study intervention and many of these patients had multiple tests. Some respondents may have interpreted “tests seen” as the number of patients with tests seen. More objective data on exposure to tests, or more detail on reported exposure (eg. asking both how many patients with results and how many tests) may have provided a more accurate classification of level of experience. However, the lower than expected test exposure may just be due to the high staff turnover.

### Mixed methods approach for future research on use of POC tests

We used a quantitative study design to assess knowledge, attitudes and practices, however, the collection of comments that respondents sometimes used to qualify their survey choices provided a deeper understanding of the main study results. The quantitative answers considered in isolation may have given a different impression. For example, whilst we found that increased experience with AChE tests led to decreased value being attributed to the test, often the same respondents who said they would not order a test also provided nuanced comments about its potential benefits. This highlights the degree of ambiguity amongst experienced respondents in their attitudes towards the test.

However, only 53% of questions had an associated comment, and thus it is possible that certain comments may preferentially highlight the views of a few individuals. We also observed that respondents who answered in favour of the test more frequently qualified their answer with a comment (67%) than when they chose not to order the test (43%), see Additional file 5: Fgure S3

Blattner et al. assessed the acceptability and effectiveness of POC testing in rural New Zealand, and carried out both a quantitative study, and a qualitative thematic analysis [8,19]. Their quantitative study demonstrated the cost effectiveness of introducing the test through ability to avoid unnecessary transfers, as well as facilitate discharge. However, the qualitative component of their research uncovered some of the challenges of introducing POC testing, such as increased workload, and the challenge of continued professional education given that “up-skilling” of doctors may be required for the interpretation of some of the available test results. These authors also commented that their depth of understanding about the impact of introducing the test would have been missed if they had relied on the quantitative results alone.

We suggest that future research on POC tests use a mixed methods approach with the development of a robust study design for its qualitative component.

## Conclusions

An AChE POC test was valued by a majority of rural doctors but it was valued less by those with greater experience of seeing test results. These unexpected findings could be related to the complex nature of the test, no decision rules and poor knowledge of the interpretation. The absence of specific education on how to interpret test results may have been a contributing factor.

We recommend that health services that want to introduce a POC AChE test provide doctors with concurrent training on how to use and interpret AChE results, and research the impact through a mixed methods approach. Such research should ideally be conducted with larger numbers, and include medical staff with a wide range of experience and include multiple primary care settings.

## Competing interests

The authors declare that they have no competing interests.

## Authors’ contributions

BNR and NB jointly designed the study. BNR carried out the methodology, analysis and drafted the manuscript. NAB helped with the analysis and revised manuscript drafts. TN advised on statistical aspects of the analysis and manuscript drafts. All authors read and approved the final manuscript.

## Pre-publication history

The pre-publication history for this paper can be accessed here:

http://www.biomedcentral.com/1472-6963/14/104/prepub

## Supplementary Material

Additional file 1: FigureS1 Flowchart showing distribution of surveys and response rate. Click here for file

Additional file 2 **Protocol for study intervention.** Appendix 1.1 - Protocol for blood testing of consenting patients according to the type of poison ingested, and whether the patient is receiving oximes. Appendix 1.2 - Time schedule for AChE blood testing. Click here for file

Additional file 3 **Survey.** Appendix 2.1 – True/false statement used to measure knowledge of biomarkers in OP poisoning (answers included). Appendix 2.2 - Questions used to measure attitudes towards AChE testing in relation to OP poisoning management and oxime therapy. Appendix 2.3 - Scenario component used to measure practice of ordering AChE in cases of severe and mild poisoning receiving and not receiving oximes. Click here for file

Additional file 4: Figure S2 Proportion of respondents ordering AChE in clinical scenarios by level of test experience; a) 0 tests, b) 1-5 tests, and c) 5-20 tests. Click here for file

Additional file 5: Figure S3 Proportion of respondents providing a clarifying comments in scenarios of a) Severe poisoning, with oxime therapy, b) Mild poisoning, without oxime therapy. Click here for file
