# Supplementary material for: Effect of acetylcholinesterase (AChE) point-of-care testing in OP poisoning on knowledge, attitudes and practices of treating physicians in Sri Lanka
Source: BMC Health Serv Res. 2014 Mar 4;14:104. doi: 10.1186/1472-6963-14-104 (PMC4015291; doi:10.1186/1472-6963-14-104)
Supplement: Additional file 1: FigureS1 — Flowchart showing distribution of surveys and response rate. [file 1472-6963-14-104-S1.pdf]

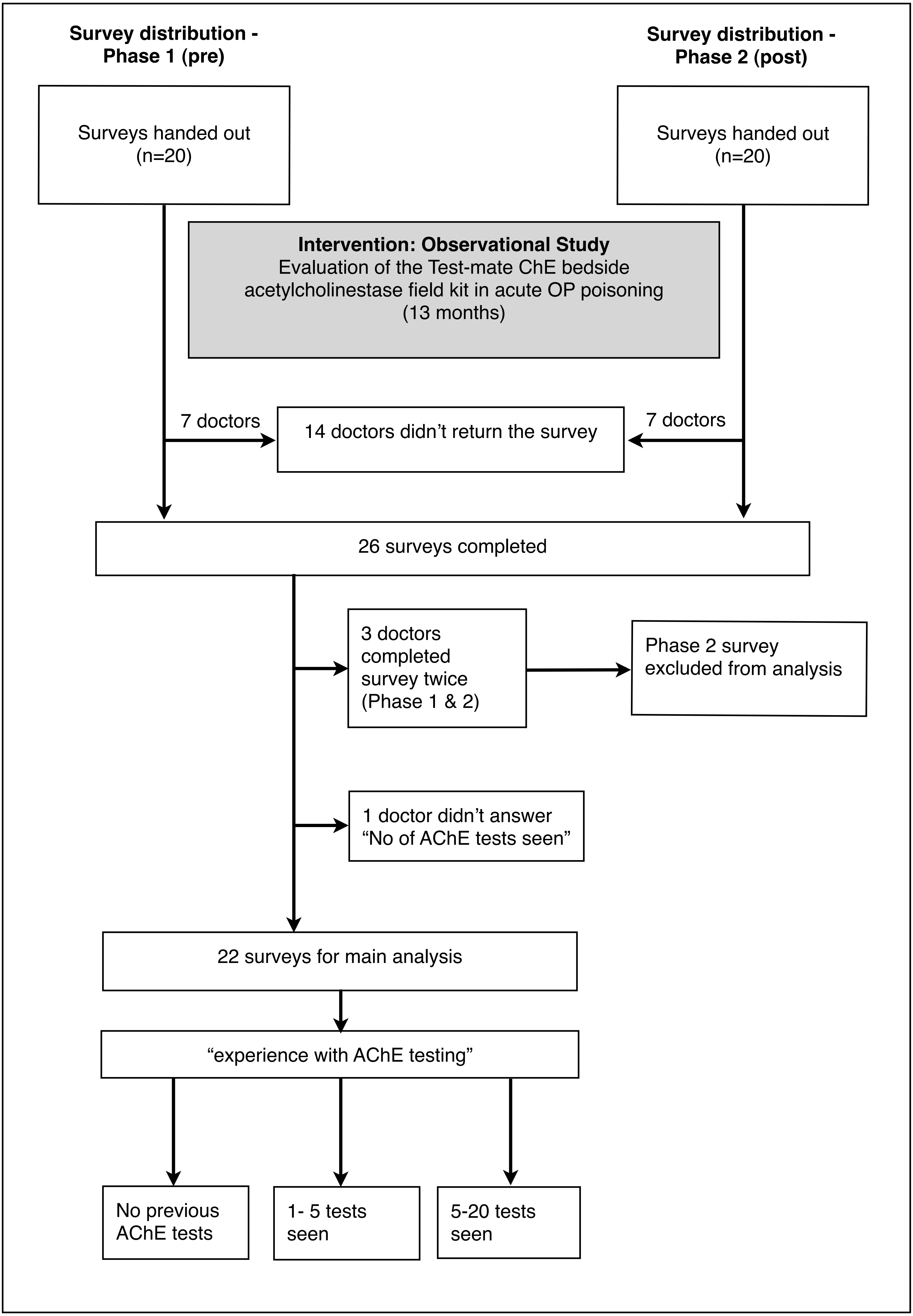
Survey distribution - Phase 1 (pre)
Survey distribution - Phase 2 (post)
Surveys handed out (n=20)
Surveys handed out (n=20)
Intervention: Observational Study
Evaluation of the Test-mate ChE bedside acetylcholinestase field kit in acute OP poisoning (13 months)
7 doctors
14 doctors didn't return the survey
7 doctors
26 surveys completed
3 doctors completed survey twice (Phase 1 & 2)
Phase 2 survey excluded from analysis
1 doctor didn't answer "No of AChE tests seen"
22 surveys for main analysis
"experience with AChE testing"
No previous AChE tests
1- 5 tests seen
5-20 tests seen
