# Supplementary figures and images for: Effect of acetylcholinesterase (AChE) point-of-care testing in OP poisoning on knowledge, attitudes and practices of treating physicians in Sri Lanka
Source: BMC Health Serv Res. 2014 Mar 4;14:104. doi: 10.1186/1472-6963-14-104 (PMC4015291; doi:10.1186/1472-6963-14-104)

## Protocol for Study Intervention

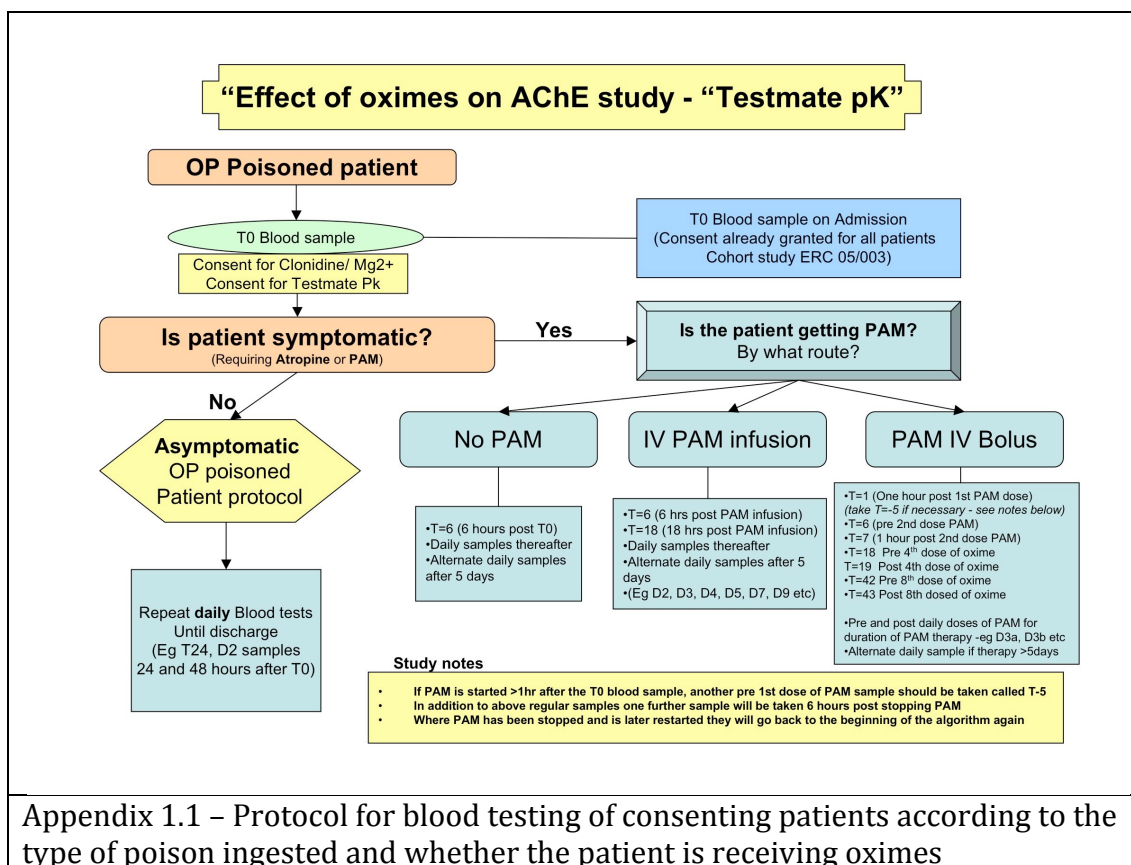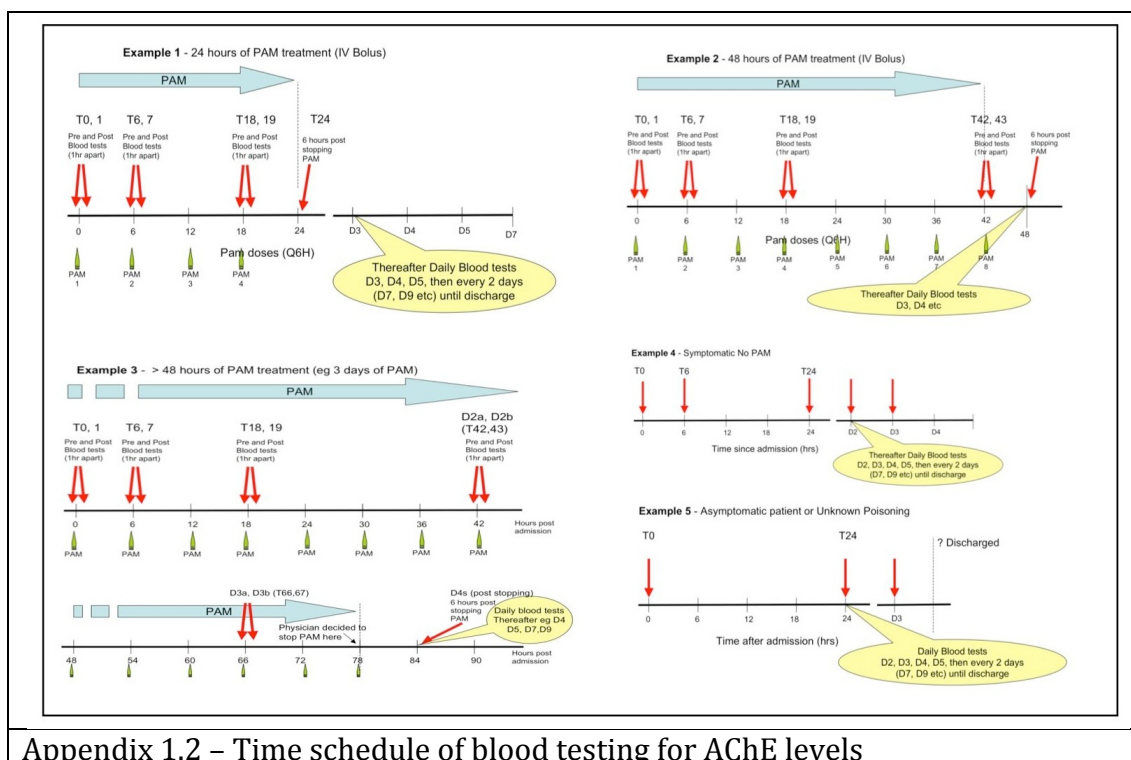

Supplement: Additional file 2 — Protocol for study intervention. Appendix 1.1 - Protocol for blood testing of consenting patients according to the type of poison ingested, and whether the patient is receiving oximes. Appendix 1.2 - Time schedule for AChE blood testing. [file 1472-6963-14-104-S2.pdf]

(0 tests)

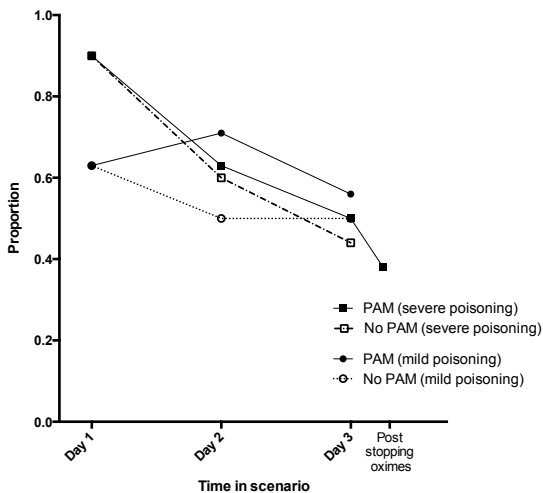

a)

(1-5 tests)

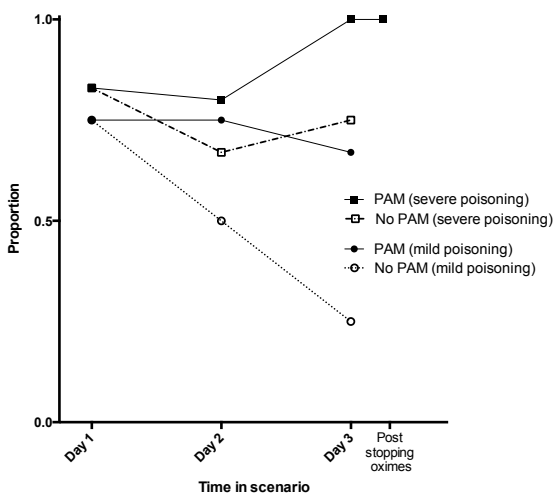

b)

(5-20 tests)

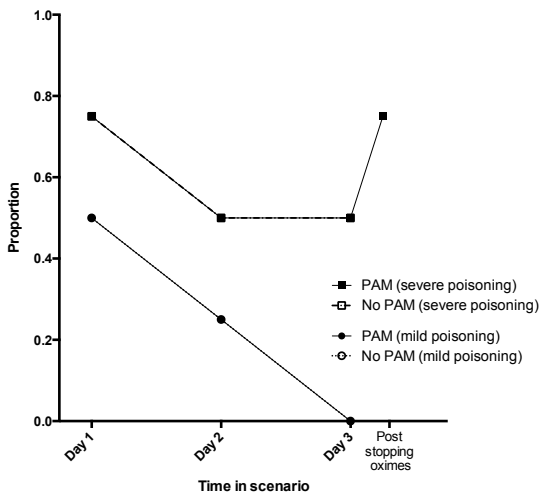

c)

Supplement: Additional file 4: Figure S2 — Proportion of respondents ordering AChE in clinical scenarios by level of test experience; a) 0 tests, b) 1-5 tests, and c) 5-20 tests. [file 1472-6963-14-104-S4.pdf]
