# Supplementary material for: Effect of acetylcholinesterase (AChE) point-of-care testing in OP poisoning on knowledge, attitudes and practices of treating physicians in Sri Lanka
Source: BMC Health Serv Res. 2014 Mar 4;14:104. doi: 10.1186/1472-6963-14-104 (PMC4015291; doi:10.1186/1472-6963-14-104)
Supplement: Additional file 3 — Survey. Appendix 2.1 – True/false statement used to measure knowledge of biomarkers in OP poisoning (answers included). Appendix 2.2 - Questions used to measure attitudes towards AChE testing in relation to OP poisoning management and oxime therapy. Appendix 2.3 - Scenario component used to measure practice of ordering AChE in cases of severe and mild poisoning receiving and not receiving oximes. [file 1472-6963-14-104-S3.pdf]

Appendix 2 – Survey

Appendix 2.1 – True/false statement used to measure knowledge of biomarkers in OP poisoning (answers included)

Appendix 2.2 - Questions used to measure attitudes towards AChE testing in relation to OP poisoning management and oxime therapy

Appendix 2.3 - Scenario component used to measure practice of ordering AChE in cases of severe and mild poisoning receiving and not receiving oximes

True / False Questions (answers)

Please circle true or false (T / F) – Don't worry, these questions are meant to be very difficult, and we don't expect everyone to get them all correct!

1) In organophosphorus (OP) poisoning;-
   a. Acetylcholinesterase in nerve synapses is inhibited — **T** / F
   b. Both Muscarinic and Nicotinic related signs and symptoms can occur — **T** / F
   c. Atropine is effective at reversing the Nicotinic effects — T / **F**
   d. Oximes act by reversing the inhibited acetylcholinesterase enzyme — **T** / F
   e. Oximes are equally effective in all types of OP poisoning — T / **F**

2) Regarding acetylcholinesterase inhibition, which of the following are true?
   a. Acetylcholinesterase that is irreversibly inhibited by OP is know as "aged" acetylcholinesterase — **T** / F
   b. Irreversible inhibition of the Muscarinic receptor can occur — T / **F**
   c. Acetylcholinesterase undergoes ageing with carbamate poisoning — T / **F**
   d. With higher OP concentrations in the blood, oximes will be less effective — **T** / F
   e. OP inhibited acetylcholinesterase can undergo spontaneous reactivation — **T** / F

3) Regarding biomarkers of exposure in OP poisoning
   a. Red blood cell acetylcholinesterase and plasma acetylcholinesterase are the same enzyme — T / **F**
   b. Both red blood cell acetylcholinesterase and plasma acetylcholinesterase can be measured in the blood — **T** / F
   c. Complete inhibition of red blood cell acetylcholinesterase is incompatible with life — T / **F**
   d. Patients with inhibition of plasma acetylcholinesterase are always symptomatic — T / **F**
   e. Plasma acetylcholinesterase is more closely correlated with clinical signs of OP poisoning than red blood cell acetylcholinesterase — T / **F**

4) On the second day post ingestion of OP poison the AChE level is **low** and shows **no change** when measured before and after a bolus of pralidoxime. This could be due to;-
   a. A high proportion of "aged" acetylcholinesterase — **T** / F
   b. Not enough atropine being given — T / **F**
   c. Not enough pralidoxime being given — **T** / F
   d. The patient has ingested a low dose of OP — T / **F**
   e. The patient took carbamates rather than OPs — **T** / F

5) Regarding pralidoxime's effect on inhibited AChE in OP poisoning
   a. Oxime therapy is more effective when given earlier than later in OP poisoning — **T** / F
   b. Poisoning with chlorpyrifos is more likely to respond than dimethoate poisoning — **T** / F
   c. The maximum inhibition of AChE is likely to occur after several days in a patient with fenthion poisoning — **T** / F
   d. 4 days following the ingestion of chlorpyrifos pralidoxime will be ineffective — T / **F**
   e. AChE inhibition in profenophos poisoning responds well to oximes — T / **F**

Survey on treatment of Organophosphorus insecticide (OP) Poisoning

Please answer the following questions by **selecting** (underline or circle) the appropriate response

General

1) Do you believe **oximes** are effective in the treatment of OP poisoning?

Yes – in all patients / Yes – in some patients / No – not in any patients / Not sure

Comments ……………………………………………………………………………..

……………………………………………………………………………………….

2a) In treating an adult male **severely symptomatic** patient (65kg) having pinpoint pupils, chest secretions, bradycardia and hypotension - What dose of intravenous pralidoxime, if any, would you prescribe? (Select from the following) ;-

None / 1g 6 hourly / 2g bolus + 500mg/hr continuous infusion / Other dose___________

2b) For what duration would you give the above dose? ;-
None / for 24 hours / for 48 hours / For other time period (comment) ___________

3a) In treating an adult male **mildly symptomatic** patient (65kg) who is getting atropine but has no respiratory or cardiovascular compromise - What dose of intravenous pralidoxime, if any, would you prescribe? (Select from the following) ;-

None / 1g 6 hourly / 2g bolus + 500mg/hr continuous infusion / Other dose___________

3b) For what duration would you give the above dose? ;-
None / for 24 hours / for 48 hours / For other time period (comment) ___________

4a) In treating an adult male **asymptomatic** patient (eg 60kg) who is not getting atropine -What dose of intravenous pralidoxime, if any, would you prescribe? (Select from the following) ;-

None / 1g 6 hourly / 2g bolus + 500mg/hr continuous infusion / Other dose___________

4b) For what duration would you give the above dose? ;-
None / for 24 hours / for 48 hours / For other time period (comment) ___________

5) Do think an acetylcholinesterase level (AChE) will be useful in helping guide treatment with oximes?
Yes / No / Not sure
Comments ……………………………………………………………………………..

……………………………………………………………………………………….

……………………………………………………………………………………….

6) If this test was available and affordable. Would it be useful in your practice of treating OP poisoning?
Yes / No
How would it help you? ……………………………………………………………………………..

……………………………………………………………………………………………………..

7) Roughly how many OP patients have you treated during your working career? (please choose)

<5	5-20	21-50	51-100	>100

8) Approximately how may AChE lab results have you seen ? (please choose)

0	1-5	5-20	21-50	51-100

Appendix 2.3 - Scenario component used to measure practice of ordering AChE

in cases of severe and mild poisoning receiving and not receiving oximes

Scenario 1 – day 1

*A 21 year old male has taken an unknown amount of an **unknown** OP poison 3 hours ago. He is symptomatic with pinpoint pupils, chest secretions, bradycardia and tachypnoea. These toxic effects appear to be responding to atropine therapy:-*

a) Would you prescribe pralidoxime to this patient? **Yes / No**

Why/ Why not?..

b) "The benefits of giving pralidoxime to this patient outweigh the risks" (**circle**):-

disagree	uncertain	agree

c) If the test were available, would you order an acetylcholinesterase (AChE) level in this patient? **Yes / No**

Why / Why not?..

d) **"**knowing this patient's admission AChE level would help his management" (**circle**) :-

disagree	uncertain	agree

please answer both Scenarios

Day 2 – 24 hours after ingestion "No oximes given"

*It is now 24 hours post ingestion of poison. He **was not** given oximes. His clinical condition has improved a little but he is still on an atropine infusion. His pupils are no longer pinpoint, his heart rate is 96, BP 120/80 and chest is clear.*

a) Would you **start** pralidoxime at this point? **Yes / No**

Why / Why not?...

b) "The benefits of giving pralidoxime to this patient ***now*** outweigh the risks" (**circle**):-

disagree	uncertain	agree

c) If the test were available, would you order an acetylcholinesterase (AChE) level at this point? **Yes / No**

Why / Why not?...

d) **"**knowing this patient's AChE level ***now*** would help his management" (**circle**) :-

disagree	uncertain	agree

Day 2 – 24 hours after ingestion – "Oximes given"

*It is now 24 hours post ingestion. He **was** given oximes. His clinical condition has improved a little but he is still on an atropine infusion. His pupils are no longer pinpoint, his heart rate is 96, BP 120/80 and chest is clear.*

a) Would you **continue** pralidoxime a further 24hours ? **Yes / No**

Why / Why not?...

b) "The benefits of giving pralidoxime to this patient ***now*** outweigh the risks" (**circle**):-

disagree	uncertain	agree

c) If the test were available, would you order an acetylcholinesterase (AChE) level at this point? **Yes / No**

Why / Why not?...

d) **"**knowing this patient's AChE level ***now*** would help his management" (**circle**) :-

disagree	uncertain	agree

Scenario 1 - day 3 (48 hours after poison ingestion)

*This patient has now been on the medical ward for 48 hours. He **has not** received **any** pralidoxime. This morning he was weaned from his atropine infusion. He has no muscarinic features or cardiovascular impairment. However, he has grade 3/5 neck muscle weakness, mild facial weakness and diminished tendon reflexes.*

a) Would you start pralidoxime at this point (48hrs) in this patient? **Yes / No**

Why / Why not?...

b) "The benefits giving pralidoxime to this patient **now** outweigh the risks" (**circle**):-

disagree	uncertain	agree

c) If the test were available, would you order an acetylcholinesterase (AChE) level at this point? **Yes / No**

Why / Why not?...

d) **"**knowing this patient's AChE level **now** would help his management" (**circle**) :-

disagree	uncertain	agree

*This patient has now been on the medical ward for 48 hours. He **has been** receiving pralidoxime. This morning he was weaned from his atropine infusion. He has no muscarinic features or cardiovascular impairment. However, he has grade 3/5 neck muscle weakness, mild facial weakness and diminished tendon reflexes.*

a) Would you continue to prescribe pralidoxime to this patient for a further 24 hours? **Yes / No**

Why / Why not?...

b) "The benefits giving pralidoxime to this patient **now** outweigh the risks" (**circle**):-

disagree	uncertain	agree

c) If the test were available, would you order an acetylcholinesterase (AChE) level at this point in this patient? **Yes / No**

Why / Why not?...

d) **"**knowing this patient's AChE level **now** would help his management" (**circle**) :-

disagree	uncertain	agree

Scenario 1 (day 3 - 6 hours later than above)

In the above scenario oximes were given initially and stopped after 48 hours. 6 hours after stopping oximes all the measured clinical parameters remained the same but the patient said he felt worse and slightly weak:-

a) Would you re-start pralidoxime at this point? **Yes / No**

Why / Why not?...

b) "The benefits giving pralidoxime to this patient **now** outweigh the risks" (**circle**):-

disagree	uncertain	agree

c) If the test were available, would you order an acetylcholinesterase (AChE) level at this point in this patient? **Yes / No**

Why / Why not?...

d) **"**knowing this patient's AChE level **now** would help his management" (**circle**) :-

disagree	uncertain	agree

Scenario 2

An 18 year old female claims to have ingested one to two mouthfuls of an unknown OP poison two hours ago. On examination she is slightly tachycardic (heart rate of 90 beats per minute) but otherwise asymptomatic.

a) Would you prescribe pralidoxime to this patient? **Yes / No**

Why/ Why not?...

b) "The benefits of treatment with pralidoxime outweigh the risks in this patient" (**circle**):-

disagree	uncertain	agree

c) If the test were available, would you order an acetylcholinesterase (AChE) level in this patient? **Yes / No**

Why/ Why not?...

d) **"**knowing this patient's admission AChE level would help her management" (**circle**) :-

disagree	uncertain	agree

please answer both Scenarios

Scenario 2A – "No oximes" (day 2 – 24 hours after ingestion)

*It is now 24 hours post ingestion of poison. This patient **has not** received any pralidoxime. Her pulse became normal soon after admission and she has remained asymptomatic thereafter.*

a) Would you **start** pralidoxime at this point? **Yes / No**

Why / Why not?...

b) "The benefits of giving pralidoxime to this patient ***now*** outweigh the risks" (**circle**):-

disagree	uncertain	agree

c) Would you discharge this patient home at this point? **Yes / No**

Why / Why not?...

d) If the test were available, would you order an acetylcholinesterase (AChE) level at this point? **Yes / No**

e) **"**knowing this patient's AChE level **now** would help her management" (**circle**) :-

disagree	uncertain	agree

Scenario 2A – "oximes given" (day 2 – 24 hours after ingestion)

*It is now 24 hours post ingestion of poison. This patient **has** received pralidoxime since admission. Her pulse became normal soon after admission and she has remained asymptomatic thereafter.*

a) Would you continue pralidoxime in this patient for a further 24 hours? **Yes / No**

Why / Why not?...

b) "The benefits of giving pralidoxime to this patient ***now*** outweigh the risks" (**circle**):-

disagree	uncertain	agree

c) Would you discharge this patient home at this point? **Yes / No**

Why / Why not?...

d) If the test were available, would you order an acetylcholinesterase (AChE) level at this point? **Yes / No**

e) **"**knowing this patient's AChE level **now** would help her management" (**circle**) :-

disagree	uncertain	agree

Scenario 2 (Day 3 - 48 hours after ingestion)

It is now 48 hours following the ingestion of poison. The patient ***has not*** *received pralidoxime. She has remained asymptomatic.*

a) Would you discharge this patient home at this point? **Yes / No**

Why / Why not?..

b) If the test were available, would you order an acetylcholinesterase (AChE) level at this point in this patient? **Yes / No**

Why / Why not?..

c) **"**knowing this patient's AChE level **now** would help her management" (**circle**) :-

disagree	uncertain	agree

It is now 48 hours following the ingestion of poison. The patient ***received*** *pralidoxime for 24 hours after admission and then it was stopped. In the second 24 hours she did not receive any medical treatment, and has remained asymptomatic.*

a) Would you discharge this patient home at this point? **Yes / No**

Why / Why not?..

b) If the test were available, would you order an acetylcholinesterase (AChE) level at this point in this patient? **Yes / No**

Why / Why not?..

c) **"**knowing this patient's AChE level **now** would help her management" (**circle**) :-

disagree	uncertain	agree

General comments about these scenarios / any complaints or things your didn't understand;-
