# Supplementary material for: Effect of acetylcholinesterase (AChE) point-of-care testing in OP poisoning on knowledge, attitudes and practices of treating physicians in Sri Lanka
Source: BMC Health Serv Res. 2014 Mar 4;14:104. doi: 10.1186/1472-6963-14-104 (PMC4015291; doi:10.1186/1472-6963-14-104)
Supplement: Additional file 5: Figure S3 — Proportion of respondents providing a clarifying comments in scenarios of a) Severe poisoning, with oxime therapy, b) Mild poisoning, without oxime therapy. [file 1472-6963-14-104-S5.pdf]

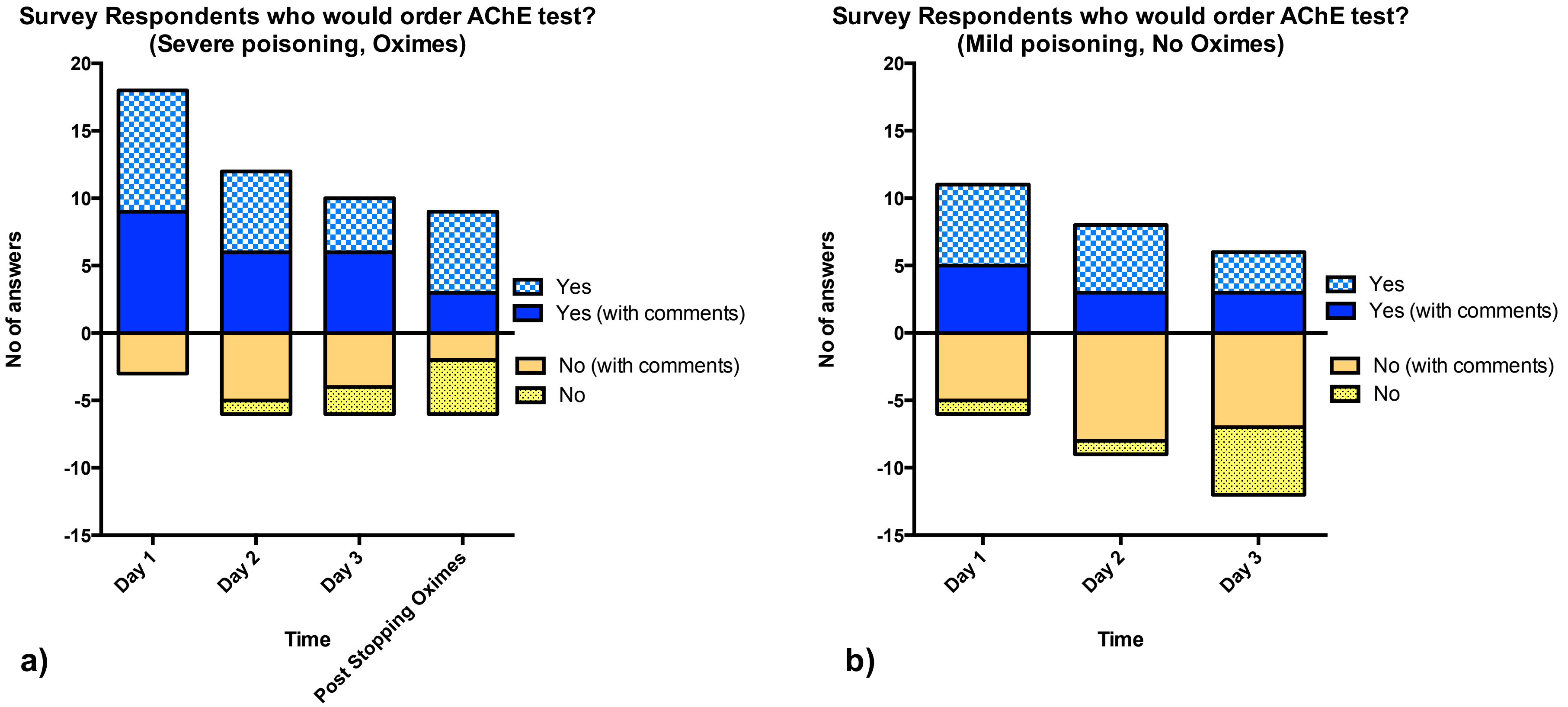

Survey Respondents who would order AChE test?
(Severe poisoning, Oximes)
No of answers
20
15
10
5
0
-5
-10
-15
Yes
Yes (with comments)
No (with comments)
No
Day 1
Day 2
Day 3
Post Stopping Oximes
Time
a)
Survey Respondents who would order AChE test?
(Mild poisoning, No Oximes)
No of answers
20
15
10
5
0
-5
-10
-15
Yes
Yes (with comments)
No (with comments)
No
Day 1
Day 2
Day 3
Time
b)
